# Personal

**Published:** 1896-09

**Authors:** 


### Personal.

Among Buffalo physicians who are enjoying or have finished their vacations we have learned of the following: Dr. John H. Pryor is in the Canada woods for a few weeks; Dr. C. C. Frederick is at Block Island; Dr. and Mrs. A. Dagenais have returned from Europe; Dr. G. A. Himmelsbach has been at Muskoka and Parry Sound; Dr. and Mrs. H. H. Bradley have made the tour around the lakes; Dr. Charles Weil is at the seashore; Dr. John Parmenter and his family are at Hamburg-on-the-Lake for the summer; Dr. Jerome Hilton Waterman, who is serving in one of the New York hospitals, is spending his vacation at home in Buffalo; Dr. William C. Krauss is absent for a fortnight, during which he attended the meeting of the American Microscopical Society at Pittsburg.

---

Dr. Edward N. Brush, superintendent of the Sheppard Asylum for the Insane, at Towson, Md., has been appointed professor of psychiatry in the Women's Medical College at Baltimore. The Journal offers its congratulations to Dr. Brush, who formerly resided in Buffalo and was an associate editor of the Journal.

---

Dr. X. O. Werder, of Pittsburg, has gone abroad for his summer vacation and will attend the International Congress of Gynecology and Obstetrics at Geneva, during the first week in September, as one of the accredited delegates from the American Association of Obstetricians and Gynecologists.

General Francis A. Walker, president of the Massachusetts Institute of Technology, received the degree of LL. D. from the University of Edinburgh at its recent convocation.

---

Dr. J. B. S. Holmes, of Atlanta, has been appointed a member of the Georgia State Medical Examining Board, to succeed Dr. J. C. Olmstead, resigned.

